# Graphemes Sharing Phonetic Features Tend to Induce Similar Synesthetic Colors

**DOI:** 10.3389/fpsyg.2017.00337

**Published:** 2017-03-13

**Authors:** Mi-Jeong Kang, Yeseul Kim, Ji-Young Shin, Chai-Youn Kim

**Affiliations:** ^1^Department of Psychology, Korea UniversitySeoul, Korea; ^2^Department of Korean Language and Literature, Korea UniversitySeoul, Korea

**Keywords:** synesthesia, grapheme-color association, phonetic property, articulatory rules, cross-linguistic approach

## Abstract

Individuals with grapheme-color synesthesia experience idiosyncratic colors when viewing achromatic letters or digits. Despite large individual differences in grapheme-color association, synesthetes tend to associate graphemes sharing a perceptual feature with similar synesthetic colors. Sound has been suggested as one such feature. In the present study, we investigated whether graphemes of which representative phonemes have similar phonetic features tend to be associated with analogous synesthetic colors. We tested five Korean multilingual synesthetes on a color-matching task using graphemes from Korean, English, and Japanese orthography. We then compared the similarity of synesthetic colors induced by those characters sharing a phonetic feature. Results showed that graphemes associated with the same phonetic feature tend to induce synesthetic color in both within- and cross-script analyses. Moreover, this tendency was consistent for graphemes that are not transliterable into each other as well as graphemes that are. These results suggest that it is the perceptual—i.e., phonetic—properties associated with graphemes, not just conceptual associations such as transliteration, that determine synesthetic color.

## Introduction

Individuals with grapheme-color synesthesia experience “colors” when viewing achromatic letters or digits. The association in each pair of the inducing grapheme and the induced color is idiosyncratic. In addition, the synesthetic color induced by a grapheme is not the same among different synesthetes. Therefore, the large individual differences in grapheme-color associations have long been considered a key characteristic in synesthesia. More recently, however, it has been recognized that there may be potential regularities of synesthetic grapheme-color association hidden behind the large individual variances (Simner et al., 2005). One of the early attempts showed that vowel characters tend to induce white, weak, or no synesthetic color (Lay, 1896; Marks, 1975; Baron-Cohen et al., 1993; Day, 2005; Smilek et al., 2007; but also see Beeli et al., 2007). Another study showed that the initial letter of a color term tends to be associated with the “color” represented by the term. For instance, “b” and “y” are typically associated with blue and yellow, respectively (Rich et al., 2005). Yet other studies have shown that letters of higher frequency tend to be associated with synesthetic colors with longer wavelengths (Herman et al., 2013) or with higher saturation (Beeli et al., 2007; Kim and Kim, 2014).

In a related vein, it has been suggested that graphemes sharing a perceptual feature tend to be associated with similar synesthetic colors (Mills et al., 2002). On one hand, some studies have suggested graphemes of a similar *shape* tend to elicit analogous synesthetic colors (Brang et al., 2011; Watson et al., 2012). On the other, studies have shown that graphemes with a similar *sound* tend to trigger similar synesthetic colors (Asano and Yokosawa, 2011, 2012; Shlyakhova, 2015). These seemingly inconsistent results regarding the determinants of synesthetic color might stem from individual differences of the synesthetes tested, which is given the limited sample sizes in most of the studies. However, it is also possible that intrinsic characteristics of the language studied in each work lead the discrepancies (Asano and Yokosawa, 2013). Those studies suggesting the importance of shape over sound tested the synesthetes “seeing colors” on Latin alphabets used by English language (Brang et al., 2011; Watson et al., 2012). English orthography has weak or inconsistent association between grapheme and sound, since a single grapheme can be mapped with multiple phonemes. For instance, grapheme “c” has several phonemes when accompanied by other graphemes (e.g., /s/ in *cite* or /k/ in *cause*, etc). In contrast, Asano and Yokosawa (2011, 2012) tested Japanese grapheme-color synesthetes on a color-matching task and found that graphemes with the same sound in two Japanese scripts (i.e., hiragana and katakana) tend to elicit similar synesthetic colors despite different shapes. Since every Japanese character maps to the same sound sequence consistently regardless of context and the two Japanese scripts are arranged in the same order based on identical phonological information, Japanese graphemes might have stronger association with sound quality than do Latin alphabets.

Some previous works have taken cross-linguistic approaches to consider the possibility that inherent differences between languages affect synesthetic grapheme-color association (Rouw et al., 2014; Shin and Kim, 2014). For example, Shin and Kim tested four Korean multilingual synesthetes who can read, write, and speak Korean, English, and Japanese and also experience synesthetic colors from scripts of those three languages. They matched their “colors” on graphemes and syllabaries of those multiple scripts. The results showed some influences of shape and meaning associated with graphemes and syllabaries in determining the synesthetic colors. However, the influence of sound was the strongest among those other factors. Namely, graphemes and syllabaries representing similar sounds tend to induce similar synesthetic colors. For example, for one synesthete, both Korean hangul syllable “
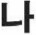
” and hiragana “
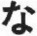
” (both sounded as /na/) induced similar synesthetic color. These results imply that the sound quality is indeed what determines synesthetic color, which is not confined to a specific language.

While these results extended the findings of Asano and Yokosawa (2011, 2012) and underscored the importance of sound as a determining perceptual factor of synesthetic color, the results could also be interpreted to result from a high-level confounding factor such as conceptual association. The pairs of inducing characters analyzed in the study of Shin and Kim, such as hangul syllable “
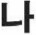
” and hiragana “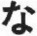,” are pronounced almost identically, and therefore retain phonetic similarity. At the same time, the character from the foreign language “
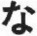
” can be transliterated into the first language “
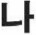
” for the Korean synesthetes. Therefore, the two characters are conceptually associated via transliteration, not just sharing an acoustic property. This alternative hypothesis is not far-fetched, since conceptual knowledge and memory have been suggested to be critical in synesthesia (Chiou and Rich, 2014). Indeed, some of our synesthetes reported potential influence of transliteration. For one, YMK said “the colors of the letters in foreign language seem to be identical to the colors of letters of the first language (L1) if the sound of the letters from a foreign language is similar to that of letters from L1.” For another, HWP described her synesthetic colors of the letters of foreign language by way of synesthetic colors of the letters from L1 such as “the color of Japanese “
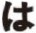
” (hiragana syllable pronounced as /ha/) is overlapped to the color of Hangul “
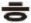
” (Korean grapheme pronounced as /h/)”. If transliterability between graphemes of different languages were the source of the previous results, it weakens the implication of the results that graphemes sharing a phonetic quality tend to induce similar synesthetic color.

This poses a serious concern because the investigation into the non-random association between a certain aspect of inducers and concurrents in synesthesia (graphemes and colors respectively in this paper) bears implications for cross-modal association between features in human languages and thoughts in general (Ramachandran and Hubbard, 2001; Treisman, 2005; Deroy and Spence, 2015). For instance, when non-synesthetic people are asked to match either rounded or sharp objects to words “bouba” and “kiki,” they consistently associate rounded contour to word “bouba” and sharp one to “kiki” more often than chance (Köhler, 1947; Werner, 1948; Werner and Wapner, 1952). Some researchers suggest that this non-arbitrary cross-modal correspondences shown in a majority of people can share similar underlying mechanism with synesthesia. That is, the intrinsic cross-modal association is on the continuum of stronger form of association experienced by synesthetes (Karwoski et al., 1942; Martino and Marks, 2001; but see also Deroy and Spence, 2013). It inversely implies that understanding synesthetic association can provide a clue for understanding cross-modal associations implicated in human language and thinking, going beyond a matter of few special individuals.

**Figure 1:**
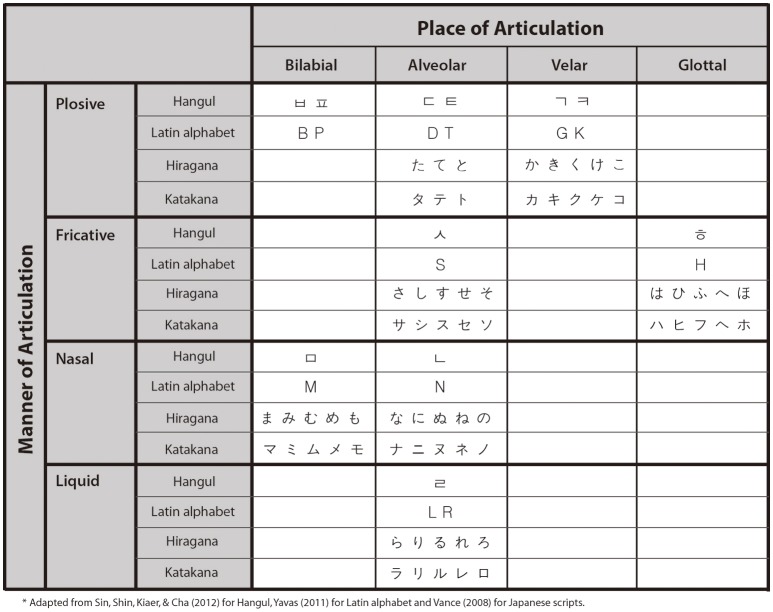
**Graphemes of multiple scripts categorized by the articulatory rules of their representative phonemes**.

Hence, in the present study, we aimed at examining whether graphemes associated with similar phonetic feature tend to induce analogous synesthetic colors even without the intervention of conceptual association. For that purpose, we categorized graphemes based on two articulatory rules of consonants—i.e., the place and the manner of articulation—reflecting basic features of sound associated with the inducing graphemes and compared the synesthetic colors of the graphemes within each category (see Figure 1).

Introducing the articulatory rules has several advantages. First, as mentioned earlier, it can rule out the confounding factor of conceptual association, since each category includes graphemes that are not transliterated with each other despite the shared phonetic quality, as well as those that are. For example, in the alveolar category, the acoustic qualities of graphemes “t” and “
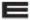
” (Korean consonant pronounced as /t/)' are transliterated with each other, whereas “t” and “
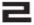
” (Korean consonant pronounced similar to /r/ or /l/)' are not. Another benefit of employing the rules is that they offer a more objective set of criteria to pair and compare graphemes from different languages, since the rules are universal classification which can be applied to every language.

We tested five Korean multilingual synesthetes on a color-matching task using graphemes from Korean (hangul), English (Latin alphabets), and Japanese orthography (hiragana and katakana) as inducing stimuli. We analyzed the matched colors in three ways. First, in the within-script analysis, we examined the similarity of the synesthetic colors induced by those graphemes whose representative sounds are classified into the same category within each script. Second, in the cross-script analysis, we investigated whether the influence of the phonetic features on synesthetic color also exists between graphemes across different scripts. Finally, we took a closer look at those graphemes categorized as the same but which cannot be transliterated into each other (e.g., both “t” and “
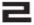
”), to examine whether they are still associated with similar synesthetic colors. This final analysis was expected to distinguish conceptual association via transliteration from perceptual similarity. Detailed methods and the results of those three analyses are described below.

## Materials and methods

### Participants

Five Korean multilingual grapheme-color synesthetes participated in the study (four females, 20–27 years of age). All participants use Korean as their first language and had studied English as their second language for about 10 years at the time of the study. They experience synesthetic colors for hangul, Latin graphemes, and Japanese syllabaries (hiragana and katakana); three of them experience colors on both hiragana and katakana, whereas two participants experience colors only on hiragana. All of them showed high consistency over multiple color matching results, and identified as synesthetes whose consistency scores are below 135 (Rothen et al., 2013; Supplementary Figure 1). All participants were classified as associators because they reported their experienced synesthetic colors appeared in their mind's eye (Dixon et al., 2004). They had normal or corrected-to-normal visual acuity. They provided written informed consent approved by Korea University Institutional Review Board (1040548-KU-IRB-15-67-A-2).

### Stimuli

Fourteen hangul, 26 Latin graphemes, and 90 Japanese syllabaries (45 hiragana and 45 katakana) were used as stimuli for synesthetic color matching task. Based on the previous work showing the similarity of synesthetic colors of graphemes and syllabaries sharing initial phonemes (Shin and Kim, 2014), we only included graphemes for hangul and Latin scripts, not syllabaries the sounds of which match those of Japanese syllabaries. Each stimulus was presented in black Arial font on the screen. The size of the stimuli differed slightly between languages due to the differences in composition and shape. Stimuli subtended a visual angle of 1.99° × 1° for hangul, 1.57° × 2.14° for Latin, 1.57° × 1.85° for Japanese graphemes, on average.

### Apparatus

Stimuli were presented on a 19-inch color-calibrated CRT monitor (1,024 × 768 resolution, 60 Hz frame rate) controlled by an Intel PC using Matlab 7.0.4 (MathWorks, Inc., 2005) and the Psychophysics Toolbox 2.54 (Brainard, 1997; Pelli, 1997). The display monitor white point was [0.295, 0.338, 41.789], in terms of the chromaticity coordinates and luminance. RGB primaries were [0.588, 0.334, 7.729], [0.286, 0.579, 29.25], and [0.168, 0.096, 4.81], respectively. Testing was conducted in a quiet and dark room in which the video monitor provided the only source of illumination.

### Procedure

**Figure 2:**
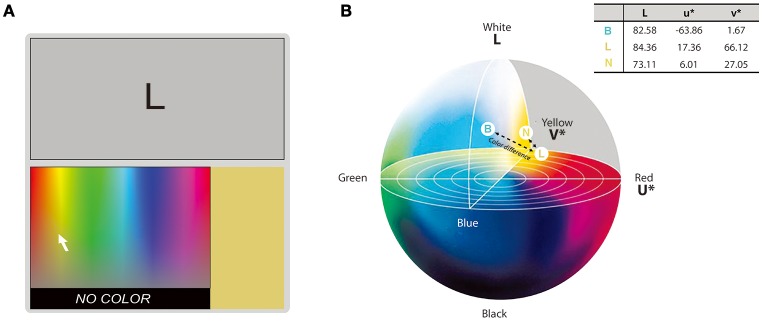
**Color matching procedures and the CVI. (A)** An example trial of the color-matching test. The synesthete selected his/her synesthetic color on the color palette at the bottom of the screen while viewing a black grapheme on top. **(B)** Examples of three matched colors on the CIELUV color space. The L, u^*^, and v^*^ color coordinates of the matched colors to each grapheme (inset) were identified and the Euclidean distance between each pair of the matched colors (dashed line) was taken as CVI. A closer distance indicates similar synesthetic colors (e.g., “L” elicits a synesthetic color more similar to “N” than to “B”).

The experimental procedures followed those in the work by Shin and Kim (2014). A modified version of the standardized Synesthesia Battery was used for a synesthetic color-matching test (Eagleman et al., 2007; Carmichael et al., 2015). The test consisted of up to four blocks of graphemes of hangul, Latin alphabets, Japanese hiragana and katakana separately. The number of blocks was varied based on the number of scripts each synesthete can use and experience colors with. In each block, individual stimuli were presented three times and the order of stimulus presentation was randomized. In each trial, one achromatic inducing stimulus was presented with a color palette at the bottom (Figure 2A). Participants were instructed to choose the induced synesthetic color from the color palette while viewing it as long as they wanted to. The brightness of the chosen color was adjustable by pressing the left- or right-arrow button on the keyboard. Participants were asked to press the “No color” button at the bottom of the color palette if they did not experience synesthetic color for the stimulus. Participants were seated 68 cm away from the monitor and their heads were stabilized using a head-chin rest.

### Data analyses

A consistency score based on the color-matching results was computed for each stimulus (Rothen et al., 2013). Following the criterion for the consistency of synesthetic color matching, only the stimuli with consistency scores below 135 were included for further analyses (For individual proportions of excluded stimuli, see Supplementary Figure 1). In addition, vowel graphemes were excluded since they tend to induce weak or white synesthetic colors, as mentioned above. Among the remaining graphemes, those that their associated phonemes are in common for all four scripts were utilized for further analyses to ensure valid comparison between scripts. For example, in the fricatives, phoneme /s/ has corresponding graphemes for all four scripts, whereas corresponding grapheme of phoneme /z/ only exist for Latin alphabets. A total of 11 hangul, 12 Latin graphemes, and 66 Japanese syllabaries (33 characters for hiragana and katakana, respectively) was used for analyses (Figure 1).

The matched RGB values of those 89 graphemes were converted into color coordinates in the CIELUV color space. The Euclidean distance between each pair of graphemes on the color space was calculated and taken as index for synesthetic color similarity, i.e., color variation index (CVI; Asano and Yokosawa, 2011, see Figure 2B).

All graphemes for the four different scripts were categorized into eight conditions based on two main articulatory rules regarding their representative sounds (Vance, 2008; Yavas, 2011; Sin et al., 2012, See Figure 1): the place of articulation (bilabial, alveolar, velar, or glottal) and the manner of articulation (plosive, fricative, nasal, or liquid). In the within-script analysis, graphemes were paired within each script. A mean CVI for each category was calculated by averaging CVI values for all within-script pairs across multiple scripts and, therefore, one CVI value for each category was extracted for each participant. The cross-script analysis was identical to the within-script analysis except that graphemes were paired between scripts. In the final analysis, graphemes in each category were divided into two groups based on transliterability to rule out the conceptual association factor, then the identical analysis method to within or cross-script analysis was applied. Baseline was defined as the mean CVI of all possible pairs of matched colors per participant irrespective of and articulatory rules (Supplementary Figure 1). A mean CVI for a condition which is smaller than the baseline indicates that the matched colors for the graphemes in the condition are located on the color space more closely, and thus, they tend to have similar synesthetic colors.

For statistical analyses, the mean CVI for each condition was compared with the baseline using one sample *t*-test. False discovery rate (FDR) analysis was applied to each participant separately in order to correct potential type-I error in multiple comparisons (Benjamini and Hochberg, 1995).

## Results

**Figure 3:**
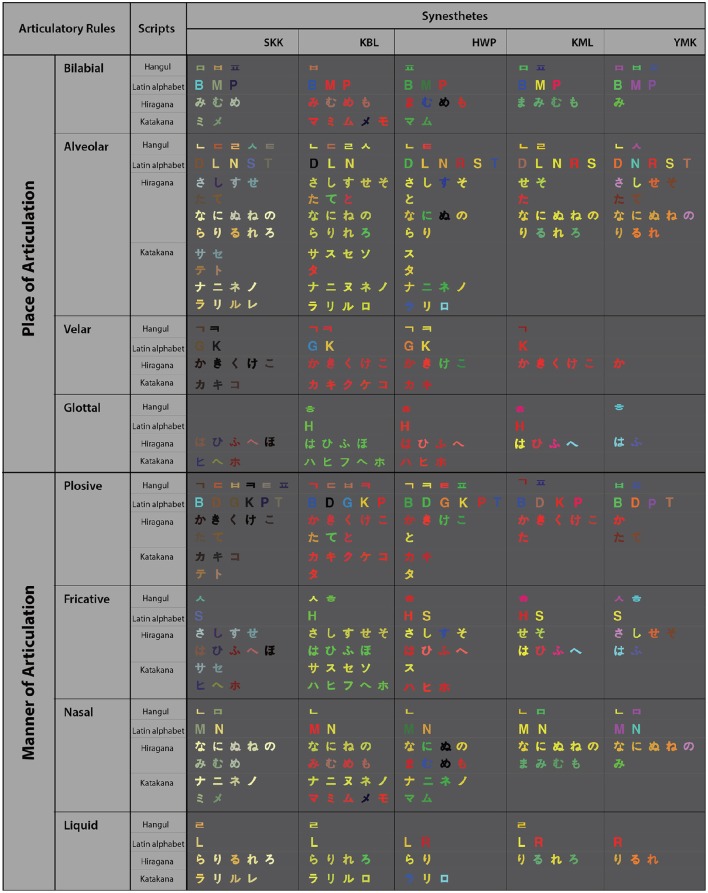
**Color matching results from all five synesthetes classified by the articulatory rules for the graphemes of multiple scripts**.

Figure 3 shows the color-matching results of all five synesthetes on all the graphemes with consonant component inducing consistent synesthetic colors, which were categorized by articulatory rules. The figure demonstrates the overall tendency for characters of the same phonetic category to take on similar synesthetic colors despite large individual differences. For example, there are more yellowish colors induced by graphemes associated with alveolar and nasal sounds across all the synesthetes tested.

### Within-script comparison

**Figure 4:**
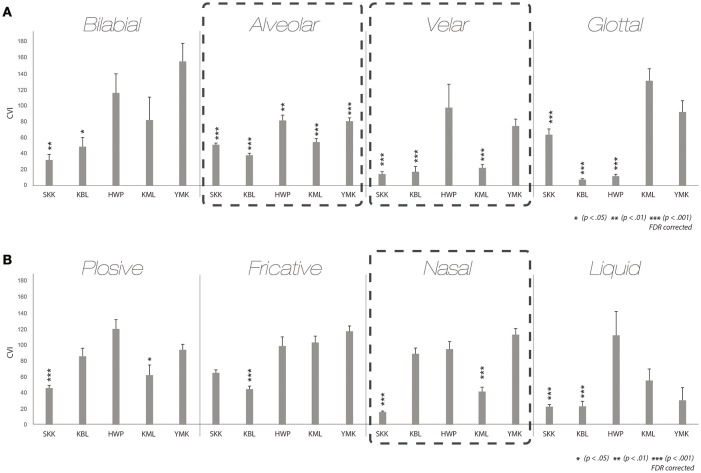
**Within-script results**. CVI results for graphemes within script categorized by the place of articulation **(A)** and the manner of articulation **(B)** for all five synesthetes. Asterisks denote the conditions of which the CVIs are significantly different from individual baseline. To be specific, the synesthetic colors of the letters in the categories denoted by the asterisks tend to be more similar than those in categories did not.

We first examined the synesthetic color similarity of pairs of graphemes in the same phonetic categories within script (Note that a phoneme associated with a grapheme is more appropriate rather grapheme itself, however, a term “grapheme” will be used for brevity from now on). The results from the CVI analyses on within-script pairs reassured us that characters belonging to the same phonetic category tended to take on similar synesthetic colors. Figure 4 shows that CVIs of the pairs of graphemes in each of the eight phonetic categories were smaller than the baseline CVI in some synesthetes^1^. This result indicates that graphemes sharing phonetic features within a language tend to induce similar synesthetic colors.

Such a tendency is more noticeable in certain categories than in others. In the alveolar category of the place of articulation, quite a few of the reported synesthetic colors experienced by the five synesthetes were similar. Moreover, the alveolar category comprised multiple graphemes in all four scripts tested (see Figures 1, 3). This was also the case for the velar and nasal categories. In the alveolar category, all five synesthetes showed statistically significant differences [SKK *t*_(238)_ = −5.826, KBL *t*_(276)_ = −19.633, *p* < 0.001, HWP *t*_(106)_ = −3.851, *p* < 0.01, KML *t*_(115)_ = −9.767, YMK *t*_(90)_ = −6.313, *p* < 0.001, *FDR corrected* for all synesthetes]. In the velar category, CVIs of three synesthetes significantly differed from the baseline CVI [SKK *t*_(13)_ = −21.278, KBL *t*_(21)_ = −12.758, KML *t*_(10)_ = −18.953, *p* < 0.001, *FDR corrected*]. When it comes to nasal category, two synesthetes showed statistically significant differences [SKK *t*_(74)_ = −27.282, KML *t*_(37)_ = −10.515, *p* < 0.001; *FDR corrected*]. These results imply that graphemes belonging to the same phonetic category tend to induce similar synesthetic colors. Moreover, such results do not seem to be solely driven by the script of their native language (hangul) but also within the scripts of the second (L2) or the third languages (L3), when the results are broken down into different scripts (Supplementary Figure 2). For a group-level analysis, we averaged CVIs of five synesthetes for each condition and compared it to the grand mean across of individual baselines (grand baseline value: 97.93), the mean CVIs of alveolar and velar categories showed statistical differences from the baseline even with the small number of participants [Alveolar: *t*_(4)_ = −4.358, *p* < 0.05; Velar: *t*_(4)_ = −3.210, *p* < 0.05; Supplementary Figure 3A]. Hence, the current results assured that graphemes sharing phonetic features tend to induce similar synesthetic colors.

### Cross-script comparison

**Figure 5:**
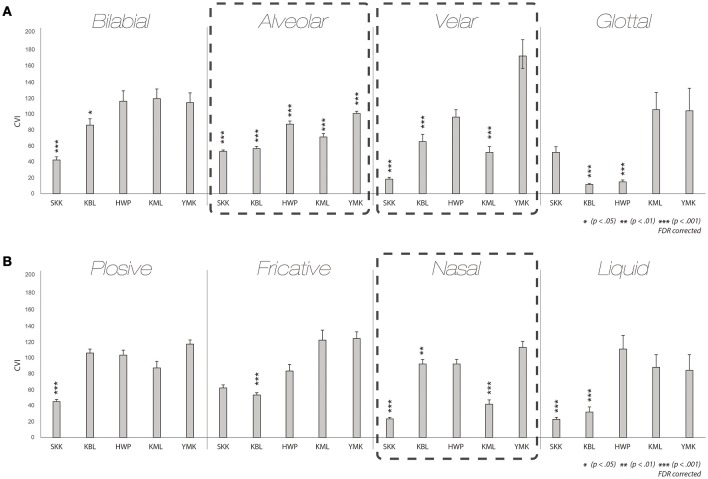
**Cross-script results**. CVI results for graphemes between scripts categorized by the place of articulation **(A)** and the manner of articulation **(B)** for all five synesthetes.

We also examined the synesthetic color similarity of pairs of graphemes across different scripts. These analyses were to investigate whether the observed effect of phonetic features of inducing graphemes on induced synesthetic colors is not confined within a script, but can be generalized across scripts of different languages. Results from the cross-script analyses were analogous to results from the within-script analyses; pairs of graphemes—one from one script and the other from another script—in the same phonetic category tended to be associated with similar synesthetic colors. The pair of graphemes categorized in the identical condition across different scripts tended to have smaller mean CVI than the baseline CVI (Figure 5).

We again focused on the alveolar, velar and nasal categories as in the within-script CVI analyses. Specifically, all five synesthetes showed statistically significant difference in the alveolar category [SKK *t*_(580)_ = −5.881, KBL *t*_(625)_ = −23.269, HWP *t*_(270)_ = −4.30, KML *t*_(114)_ = −6.967, YMK *t*_(118)_ = −4.574, *p* < 0.001, *FDR corrected*]. Three synesthetes showed statistically significant difference both in the velar and in the nasal conditions [Velar: SKK *t*_(40)_ = −19.569, KBL *t*_(68)_ = −4.480, KML *t*_(16)_ = −6.530, Nasal: SKK *t*_(155)_ = −36.067, *p* < 0.001, KBL *t*_(135)_ = −3.342, *p* < 0.01 KML *t*_(39)_ = −11.197, *p* < 0.001, *FDR corrected*]. These results imply that the influence of phonetic feature of inducing graphemes on the induced synesthetic colors is not limited within a script. Results also showed that the relationship between phonetic feature and synesthetic color is not limited between scripts of the same language (e.g., hiragana and katakana) either (Supplementary Figure 4). When we conducted the group-level analysis, the mean CVI of the alveolar category was significantly different from the mean of baseline [*t*_(4)_ = −3.283, *p* < 0.05; Supplementary Figure 3B]. Therefore, these results suggest that phonetic properties associated with graphemes are, in general, an important determinant of their synesthetic colors.

### Comparison factoring out transliterability

**Figure 6:**
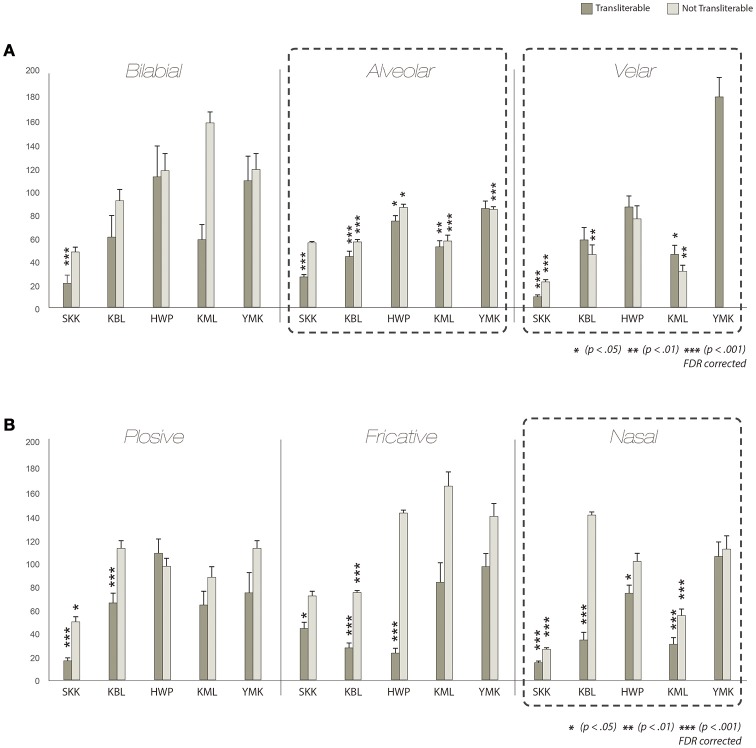
**Results factoring out transliterability**. CVI results for graphemes categorized by the place of articulation **(A)** and the manner of articulation **(B)**, according to whether consonants between the pairs of scripts are transliterable or not. Glottal in the place of articulation and liquid in the manner of articulation are excluded in the analysis, since those categories consist of only transliterable consonants. The dark gray bars indicate the transliterable pairs, whereas the light gray bars indicate those that are not. The pairs of graphemes that are not transliterable tended to have smaller mean CVI than individual baseline CVI as well as those pairs of graphemes that are transliterable.

Results from the within- and cross-script CVI analyses showed that graphemes (their associated phonemes, indeed) of the same phonetic feature tend to induce similar synesthetic colors, which leads us to conclude that phonetic feature is an important determining factor of synesthetic color. However, it is still possible that conceptual association between graphemes within the same phonetic category such as transliterability, not the acoustic feature itself, might base the finding. Therefore, we divided graphemes within each phonetic category into two subgroups: one includes those that are conceptually associated and transliterable based on readily recognizable sound similarity, and the other contains those of which phonetic similarity is not readily recognizable and thus not transliterable. For example, “t” and “
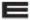
” belong to the former sub-category, whereas “t” and “
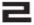
” belong to the latter one, though both pairs belong to the alveolar category. Figure 6 shows the results from the subdivided CVI analyses. Some synesthetes (e.g., SKK) showed a tendency toward smaller mean CVI in the pairs of graphemes that are transliterable to one another (dark gray bars) than in the pairs of graphemes that are not transliterable to one another (light gray bars). These results suggest that the conceptual association based on transliterability between graphemes might play a role in induced synesthetic color similarity. More relevant to our current purpose, however, those pairs of graphemes that are not transliterable tended to have smaller mean CVI than the baseline CVI as well as those pairs of graphemes that are transliterable in both the place of articulation (Figure 6A), and the manner of articulation (Figure 6B) categories. Specifically, in the alveolar category, three out of the five synesthetes showed smaller mean CVI both in pairs of graphemes that are transliterable and in those that are not, and one synesthete (YMK) showed smaller mean CVI even only in pairs of graphemes that are not transliterable [KBL T: *t*(146) = −15.485, NT: *t*(478) = −18.456, *p* < 0.001, HWP T: *t*(63) = −3.260, NT: *t*(206) = −3.136, *p* < 0.05, KML T: *t*(28) = −4.524, *p* < 0.01, NT: *t*(85) = −5.428, *p* < 0.001, YMK NT: *t*(94) = −4, 324, *p* < 0.001, *FDR corrected*]. In the velar category, two synesthetes had smaller mean CVI both in pairs of graphemes that are transliterable and in those that are not and one synesthete had smaller mean CVI only in pairs of graphemes that are not transliterable [SKK T: *t*(8) = −18.936, NT: *t*(31) = −16.029, *p* < 0.001, KML T: *t*(10) = −3.988, *p* < 0.05, NT: *t*(5) = −8.681, *p* < 0.01, KBL NT: *t*(46) = −4, 029, *p* < 0.01, *FDR corrected*]. In the nasal category, two synesthetes showed smaller mean CVI only in pairs of transliterable graphemes but two synesthetes showed smaller mean CVI both in pairs of graphemes that are transliterable and in those that are not [KBL T: *t*(67) = −18.770, *p* < 0.001, HWP T: *t*(46) = −3.146, *p* < 0.05, SKK T: *t*(77) = −38.777, NT: *t*(77) = −24.884, KML T: *t*(19) = −11.555, NT: *t*(20) = −6.437, *p* < 0.001, *FDR corrected*]. When we conducted the group-level analysis, the mean CVI of the alveolar category was significantly different from the mean of baseline both in pairs of graphemes that are transliterable and in those that are not [Alveolar T: *t*(4) = −3.212, NT: *t*(4) = −3.331, *p* < 0.05, *FDR corrected;* Supplementary Figure 3C]. These results are striking in that the phonetic similarity of non-transliterable graphemes was not necessarily recognizable, but nonetheless, those graphemes were associated with similar synesthetic colors. These results highlight the importance of phonetic properties as a determining factor of synesthetic color.

## Discussion

By exploiting such articulatory rules as the place and the manner of articulation, the current results show that graphemes associated with a shared phonetic feature tend to induce similar synesthetic colors. This was the case not just within a script, but also across multiple scripts of different languages. It is one step further from the previous findings (Asano and Yokosawa, 2011, 2012; Shin and Kim, 2014) toward verifying the importance of sound as a determining factor of synesthetic color. Moreover, our results showed that graphemes of the same phonetic category tend to be associated with similar synesthetic colors, even though they are not transliterable into each other. These results rule out the possibility that similarity of synesthetic colors induced by graphemes of shared phonetic feature is not necessarily based on conceptual association between transliterated graphemes of different languages. Taken together, the results of the current work confirm that it is the perceptual– in this case, the phonetic—feature of the graphemes, not just the conceptual linkage of the graphemes that determines synesthetic color.

We are not the first to make use of the articulatory rules to analyze inducing stimuli in studying synesthesia. A handful of studies have paid attention to the phonetic categorization in phoneme-inducing types of synesthesia. For example, a case study of lexical-gustatory synesthete JIW showed that a particular phonetic feature can be associated with a synesthetic experience of a specific taste category (Bankieris and Simner, 2014). In the study, the researchers categorized the phonetic features of a number of synesthesia-inducing words, including the place and the manner of articulation. Induced sensations were also analyzed based on five basic tastes: sweet, sour, bitter, salty, and umami. Results showed that the intensity of JIW's synesthetic taste of umami was correlated with the manner of articulation of the inducing words, and that the intensity of sour taste was predicted by the place of articulation. In other studies, concerning phoneme-color synesthesia, a vowel's place of articulation along the front-back dimension was shown to be correlated with lightness of synesthetic color (Jacobsen, 1962; Marks, 1975). That is, vowels articulated at the front are more likely to trigger lighter synesthetic colors. These studies imply that phonetic features can be influential factors in the types of synesthesia where phoneme induces synesthetic experience.

Phonetic categorization has also been applied in studying grapheme-color synesthesia. Moos and colleagues have reported systematic association between phonetic features of vowel sounds and the chromaticity and luminance of the colors participants selected when *hearing* sounds (Moos et al., 2014). This finding is in line with the earlier studies showing correlations between sound and color (Jacobsen, 1962; Marks, 1975). Interestingly, such relationships were stronger and more consistent in grapheme-color synesthetes who experience colors when *viewing* achromatic letters and digits compared to non-synesthetes, which implies interplay between phonetic and graphemic information. Our results further demonstrate that even the phonetic features implied in visually presented inducing characters have an effect on induced synesthetic colors, despite the lack of physical acoustic information. Most of the grapheme-color synesthetes we tested did not experience colors when hearing sounds, with the sole exception of HWP. Hence, the current results imply the involvement of phoneme processing even during grapheme processing, suggesting the coexistence of acoustic-phonetic and visual-graphemic influences in synesthesia (Simner et al., 2005; Simner, 2007). The current results are also in line with the recent finding that color experienced by grapheme-color synesthetes when viewing inducing graphemes is perceptually comparable to the color recalled from memory when hearing spoken graphemes (Arnold et al., 2012). These results might bear implications in more general context of language processing suggesting visual graphemes automatically activates phonological representations and auditory phonemes automatically activates visual graphemic information (Berent and Perfetti, 1995; Stone et al., 1997).

The grapheme-phoneme interaction is supported by the experimental evidence showing that the brightness and saturation of synesthetic colors correlated positively with the frequency of the inducing graphemes in spoken language (Kim and Kim, 2014). Indeed, the relationship between grapheme frequency and synesthetic color has been of enduring interest in synesthesia literature (Lewand, 2000; Beeli et al., 2007; Watson et al., 2012; Herman et al., 2013; van Leeuwen et al., 2015). In this vein, it is noteworthy that in the current results, the three phonetic categories that showed the strongest tendency of synesthetic color similarity, alveolar, velar and nasal, are the ones with relatively higher frequency of use (Hangul: Shin, 2011, Latin: Denes and Pinson, 1993, Japanese graphemes: Chikamatsu et al., 2000 for letter frequency usage in each scripts). Graphemes in these categories might have developed stronger associations with synesthetic colors in a specific fashion through frequent usage in speech. Hence, these results can be interpreted as additional evidence showing the importance of sound as determining factor of synesthetic color.

In a previous work of our group, sound was identified as the most prominent determining factor of synesthetic color (Shin and Kim, 2014). The current work further extended the previous finding by considering articulatory rules as means to examine the acoustic, phonetic influence on synesthetic colors. However, the influence of the other perceptual feature, shape for example, can still be of interest, despite the current focus on sound as determinant of synesthetic colors. Therefore, in an additional analysis, we classified graphemes of each of the three categories that we focused (alveolar, velar, and nasal) into two sub-categories based on their shape similarity, computed the CVIs of pairs of graphemes for each sub-categories and compared them to the baseline. The results showed no evidence of additive effect of shape to the effect of sound for both within- and cross-script comparisons. In other words, graphemes of similar shapes within the three phonetic categories didn't induce more similar synesthetic colors than did graphemes of dissimilar shapes.

Despite the regularity of synesthetic color association with inducing graphemes based on the phonetic features, the current results still demonstrate large individual differences. Some of the five synesthetes showed a stronger tendency to experience similar synesthetic colors from graphemes sharing phonetic features than did others. For example, SKK and KBL showed smaller CVI than the baseline in most categories of the place and the manner of articulation (see Figures 4–6), whereas YMK showed no such strong tendency. This difference does not seem to be related to the degree of fluency of the second and third languages, as suggested in previous studies (Blair and Berryhill, 2013; Mroczko-Wąsowicz and Nikolić, 2013). According to the pre-test questionnaires those synesthetes completed, KBL reported high fluency in Japanese comparable to that of his first language, Korean. In contrast, SKK reported low fluency of Japanese (Table 1 in Shin and Kim, 2014). Since KBL and SKK were the two of the five synesthetes who showed the strongest pattern of association between the sound of the grapheme and the induced synesthetic color, the fluency of Japanese does not seem to be the main cause for the effect. It is noteworthy that KBL and SKK were the two of the five synesthetes who showed the highest consistency in hangul color-matching. They also showed high consistency scores overall in Latin-alphabet and Japanese color-matching. In contrast, YMK, whose consistency score was the lowest among the five synesthetes, showed the weakest pattern of association between the sound of grapheme and the induced synesthetic color (Supplementary Figure 1). Hence, the non-random relationship between grapheme sound and color seems more apparent in synesthetes whose synesthetic association is stronger. It should be noted, however, that there are other potential factors behind the weaker association between graphemes sharing phonetic properties and synesthetic colors in YMK. In a future work, it will be useful to scrutinize other factors with the larger number of synesthetes to put the current results in a more generalized context.

Our results are limited in that they are based only on associator-type synesthetes (Dixon et al., 2004) who report “seeing” colors in their mind's eye. The brains of associators seem to show distinct functional (van Leeuwen et al., 2011), anatomical (Rouw and Scholte, 2007, 2010; Weiss and Fink, 2009, but see also Hupé et al., 2012) and oscillatory (Cohen et al., 2015, but see also Gebuis et al., 2009) characteristics different from those of projector-type synesthetes, although these claims remain controversial. Whether the degree of association between a feature of an inducing grapheme and the induced synesthetic color differs between different types of synesthetes has not been studied extensively yet; one study showed that the association between sound of an inducing grapheme and induced synesthetic color is observed in both associators and projectors (Asano and Yokosawa, 2011). Another study, however, found the association between the visual shape of an inducing grapheme and induced synesthetic color stronger in projectors than in associators (Brang et al., 2011). While we remain agnostic about the type-dependency of synesthetic association, the current results underscore that the rules of synesthetic association resemble those of multisensory association in the non-synesthetic population (Ward, 2009). Psychophysical studies on non-synesthetic individuals have reported cross-modal association between sound qualities and visual qualities, such as loudness and brightness (Wicker, 1968; Bond and Stevens, 1969; Marks, 1974), and pitch and hue (Simpson et al., 1956, see Spence, 2011 for a review). These findings are reminiscent of our results showing non-random association between graphemes sharing phonetic qualities (i.e., belonging to the same phonetic category) and induced synesthetic color.

This last point can be interpreted in the context of the two competing theories of neural mechanisms of grapheme-color synesthesia. The linkage between sound and color in grapheme-color synesthesia suggests the involvement of the multimodal cortical region, seemingly supporting disinhibited feedback from the multimodal region to the color-selective region (Grossenbacher and Lovelace, 2001). The current findings, however, are not incompatible with the updated version of the hyper-connectivity hypothesis, which extends the previously suggested hyper-connectivity between the visual word form area (VWFA) and the color-selective region (V4). Our findings can be construed by way of global hyperconnectivity, not just limited to the visual cortical regions (Hänggi et al., 2011; Hupé et al., 2012). They can also be explained by an intrinsic connection between visual color selective area V4 and the auditory cortex, which was found in grapheme-color synesthetes but not in non-synesthetic controls by using resting-state connectivity analyses (Dovern et al., 2012).

In summary, we compared the synesthetic color similarity of graphemes over multiple scripts categorized the same based on phonetic rules. Results showed non-random association between the phonetic features of inducing graphemes and the induced synesthetic color. Results further demonstrated that this association was not mainly driven by conceptual associations.

## Author contributions

CK and JS conceived the study. CK and MK designed the study. MK and YK collected and analyzed the data. MK, YK, JS, and CK wrote the paper. MK and YK contributed equally to this work.

## Funding

This work was supported by NRF-2013R1A1A1010923 from the Basic Science Research Program through the National Research Foundation of Korea (NRF) funded by the Ministry of Science, Information and Communications Technologies (ICT) and Future Planning.

### Conflict of interest statement

The authors declare that the research was conducted in the absence of any commercial or financial relationships that could be construed as a potential conflict of interest.

## Footnotes

^1^When we took the grand mean across all participants as group baseline (97.93) and performed statistical test, the results were identical to the current results.
