# Supplementary figures and images for: Graphemes Sharing Phonetic Features Tend to Induce Similar Synesthetic Colors
Source: Front Psychol. 2017 Mar 13;8:337. doi: 10.3389/fpsyg.2017.00337 (PMC5346570; doi:10.3389/fpsyg.2017.00337)

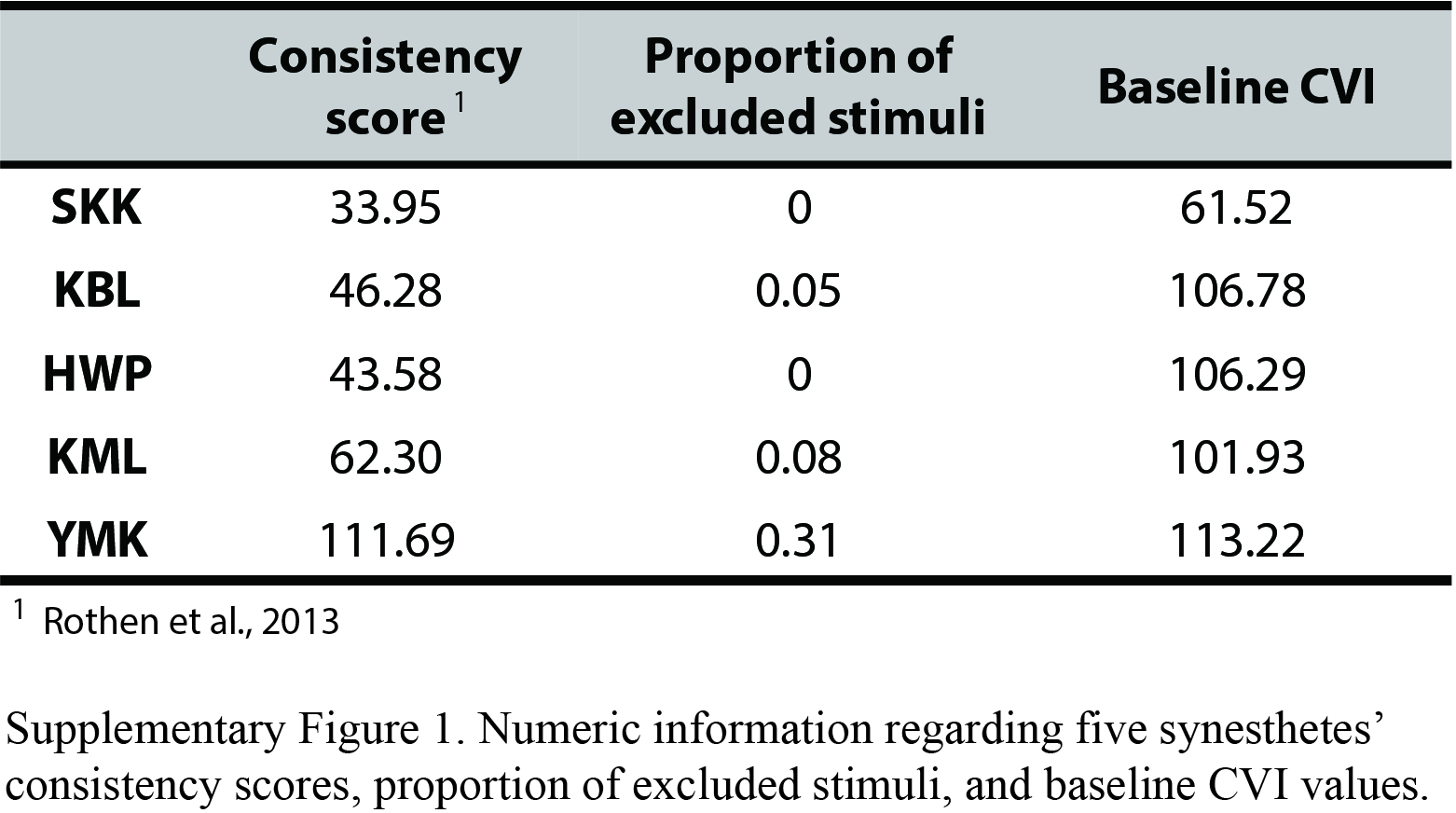

Supplement: Supplementary file 1 [file Image1.JPEG]
